# Reexamining the effects of gestational age, fetal growth, and maternal smoking on neonatal mortality

**DOI:** 10.1186/1471-2393-4-22

**Published:** 2004-12-01

**Authors:** Cande V Ananth, Robert W Platt

**Affiliations:** 1 Division of Epidemiology and Biostatistics, Department of Obstetrics, Gynecology, and Reproductive Sciences, UMDNJ-Robert Wood Johnson Medical School, New Brunswick, NJ 08901-1977, USA; 2 Departments of Pediatrics, and of Epidemiology and Biostatistics, McGill University, Montreal, Canada

## Abstract

**Background:**

Low birth weight (<2,500 g) is a strong predictor of infant mortality. Yet low birth weight, in isolation, is uninformative since it is comprised of two intertwined components: preterm delivery and reduced fetal growth. Through nonparametric logistic regression models, we examine the effects of gestational age, fetal growth, and maternal smoking on neonatal mortality.

**Methods:**

We derived data on over 10 million singleton live births delivered at ≥ 24 weeks from the 1998–2000 U.S. natality data files. Nonparametric multivariable logistic regression based on generalized additive models was used to examine neonatal mortality (deaths within the first 28 days) in relation to fetal growth (gestational age-specific standardized birth weight), gestational age, and number of cigarettes smoked per day. All analyses were further adjusted for the confounding effects due to maternal age and gravidity.

**Results:**

The relationship between standardized birth weight and neonatal mortality is nonlinear; mortality is high at low z-score birth weights, drops precipitously with increasing z-score birth weight, and begins to flatten for heavier infants. Gestational age is also strongly associated with mortality, with patterns similar to those of z-score birth weight. Although the direct effect of smoking on neonatal mortality is weak, its effects (on mortality) appear to be largely mediated through reduced fetal growth and, to a lesser extent, through shortened gestation. In fact, the association between smoking and reduced fetal growth gets stronger as pregnancies approach term.

**Conclusions:**

Our study provides important insights regarding the combined effects of fetal growth, gestational age, and smoking on neonatal mortality. The findings suggest that the effect of maternal smoking on neonatal mortality is largely mediated through reduced fetal growth.

## Background

Birth weight is arguably one of the strongest predictors of infant survival, yet its role as a causal predictor of mortality is poorly understood [1]. This is at least partly because low birth weight (<2,500 g) is a construct of two intricately intertwined components: preterm delivery and reduced fetal growth, or both. Our lack of understanding of the complex relationship among birth weight, gestational age and perinatal mortality stems from mixing etiologically distinct pathways to mortality, namely effects chiefly due to fetal maturity (*i.e*., gestational age) versus those related to fetal growth.

Disentangling the intricate pathways of gestational age and fetal growth to neonatal mortality gets even more complicated by the consideration of a third factor – maternal smoking during pregnancy. Smoking has been clearly associated with poor reproductive outcomes, including increased risk of preterm birth, stillbirth, and a range of other outcomes [2-6]. Recent studies suggest a more direct and stronger association between maternal smoking and "fetal growth" (birth weight-for-gestational age) than with preterm delivery [7], suggesting that the effect of smoking on mortality may be largely mediated through restricted fetal growth rather than preterm delivery.

To better understand the relationship among these indices of "fetal wellbeing", we examined neonatal mortality in relation to standardized birth weight (*i.e*., z-score birth weight), gestational age, and smoking during pregnancy. We applied nonparametric logistic regression based on generalized additive models to examine neonatal mortality in relation to 3 factors.

## Methods

### Cohort composition of United States live births

Data for this study were derived from the 1998–2000 United States vital statistics data files (live births linked to infant deaths), assembled by the National Center for Health Statistics of the Centers for Disease Control and Prevention [8]. The analysis was restricted to singleton live births, with neonatal mortality defined as deaths within the first 28 days. Gestational age assignment in these data are predominantly based on self-reported last menstrual period, with a small fraction (<5%) based on the clinical estimate [9]. Further, the National Center for Health Statistics imputed missing gestational ages in these data files prior to release of the data [10].

Information on smoking during pregnancy was available in two forms on the vital statistics data: one as an indicator variable (yes or no), and the other as a continuous variable denoting the number of cigarettes smoked per day during pregnancy. Both of these smoking measures were based on maternal self-report. Information on smoking patterns across different trimesters in pregnancy was not available on the vital records.

Fetal growth was defined as birth weight-for-gestational age, and was expressed as gestational age-specific birth weight z-score. This z-score construct is interpreted as units of standard deviations from the population-specific mean birth weight at each gestational age. The z-score or standardized birth weight follows a Gaussian distribution with mean 0 and variance 1.

In addition to the full analysis, we also examined in a sub-analysis the impact of implausible birth weight/gestational age combinations on overall results. These implausible birth weight/gestational ages were identified if infants' birth weights were outside the gestational age-specific birth weight cutoffs [11]. This was done to examine the impact of largely apparent gestational age errors (*e.g*., infant delivered at 26 weeks with a birth weight of 4,000 g) on neonatal mortality.

### Data exclusions

There were 11,677,103 singleton live births from which we excluded infants with missing birth weight or gestational age (n = 237,433), and birth weight <500 g or gestational age <24 weeks (n = 28,732). Since smoking data was not reported on vital statistics in California, Indiana, New York state, and South Dakota [8], births from these states were also excluded (n = 1,326,841). After all exclusions, 6,117,808 singleton live births remained for analysis.

### Statistical analysis

We examined the distributions of z-score birth weight, gestational age, and number of cigarettes smoked per day, and compared these distributions between the two groups of neonatal mortality. Neonatal mortality was then modeled using nonparametric logistic regression based on generalized additive models [12]. GAM is one modeling approach that makes no assumptions about the functional form of the exposure-disease relationship except for smoothness, *i.e*., continuity of the dose-response function and its low-order derivatives [13]. When combined with more traditional modeling approaches, GAMs are powerful graphical tools that can provide interesting insights about complex relationships. While polynomial models [14] could be used to the same end as GAM-based approaches, such models result in restricted shapes, especially at the tail of the distribution, and may not be as statistically efficient as nonparametric models. Therefore, these models were not considered.

All regression models were adjusted for the confounding effects due to maternal age and gravidity (*i.e*., number of pregnancies). We examined the associations between neonatal mortality and each of the 3 factors z-score birth weight, gestational age, and number of cigarettes smoked per day separately. We then fit a full model for mortality after forcing all 3 predictors (in addition to the confounders) as described in the Appendix [see additional file 1]. The independent effect of each of these 3 factors on neonatal mortality was assessed by comparing the residual deviances [12] between nested models (*i.e*., comparing the residual deviances from a full model to a model without the predictor). Under the large sample assumption, the deviance has an approximate chi-square distribution, with degrees-of-freedom for the test being the difference in the degrees of freedom between the nested models being compared. We also examined the distribution of partial residuals [12] from fitting the model to assess departures from adequate fit.

In addition, we tested for all possible two-factor interactions between the predictors. Although all interactions were statistically significant (owing to the large study size), none provided any additional insights that were different from a model that contained no interaction terms. Therefore, we did not consider assessing two-way interactions in the analysis.

All statistical analyses were performed in S-Plus (Insightful Corporation, Seattle WA) version 6.2 on the UNIX (Sun Microsystems, Inc: Palo Alto, CA). Nonparametric logistic regression models were fit using the *gam( ) *function based on the it loess scatterplot smoother [14], using the default span of 50%. Given the large size of the study, small changes in the span resulted in statistically significant improvement in the fit, while offering very little clinical insight. Thus, we resorted to the default span.

## Results

**Figure 1:**
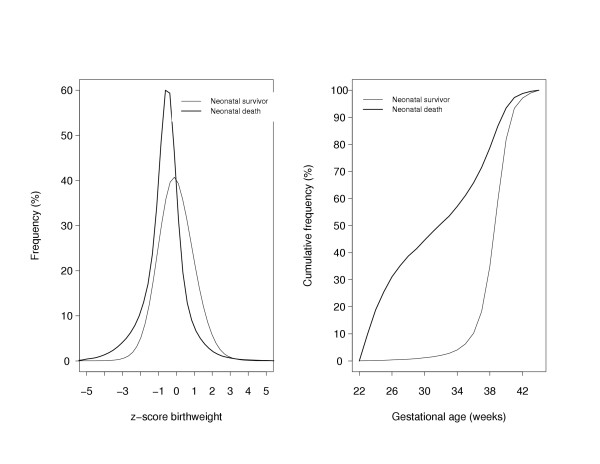
Distributions of z-score birth weight (left panel) and gestational age (right panel) among neonatal deaths (thick line) and neonatal survivors (thin line).

The overall neonatal mortality rate was 2.4 per 1,000 live births. The distribution of z-score birth weight among infants that died during the neonatal period was shifted more towards lower standardized birth weights than among those that survived the neonatal period (Fig 1, left panel). Infants who died during the neonatal period were delivered earlier than those that survived the neonatal period (Fig 1, right panel). Surviving infants weighed, on average, 1,582 g more compared with those who died during the neonatal period (P < 0.0001; Table 1). Likewise, infants who died during the neonatal period were delivered, on average, 7 weeks earlier than those who survived the neonatal period (P < 0.0001). The proportion of mothers that smoked during their pregnancy was higher among infants that died during the neonatal period (19.1%) compared with those that survived the neonatal period (16.5%; P < 0.0001).

**Table 1:** Distributions of birth weight, gestational age, and maternal smoking in relation to neonatal survival status

	**Neonatal survivors**	**Neonatal deaths**
Total events	10,084,106	27,355
Maternal age (years)†	27.0 (6.2)	26.4 (6.7)
Primigravida	33.2%	34.1%¶
Birth weight (grams)†	3,347 (572)	1,765 (1,145)
Birth weight <2,500 grams	6.1%	69.6%
Birth weight <1,500 grams	1.1%	62.3%
z-score birth weight†	0.00 (1.00)	-0.62 (1.07)
Gestational age (weeks)†	38.9 (2.3)	31.3 (6.6)
Delivered <37 weeks	10.3%	67.9%
Delivered <34 weeks	3.0%	63.6%
Delivered <32 weeks	1.7%	61.2%
Smoking during pregnancy		
Smokers	16.5%	19.1%
Cigarettes smoked/day‡	11 (1–40)	15 (1–40)

† Data expressed as mean (standard deviation).

‡ Data expressed as median (range) among all smokers.

¶ P-value <0.01. For all other comparisons, P < 0.0001.

**Figure 2:**
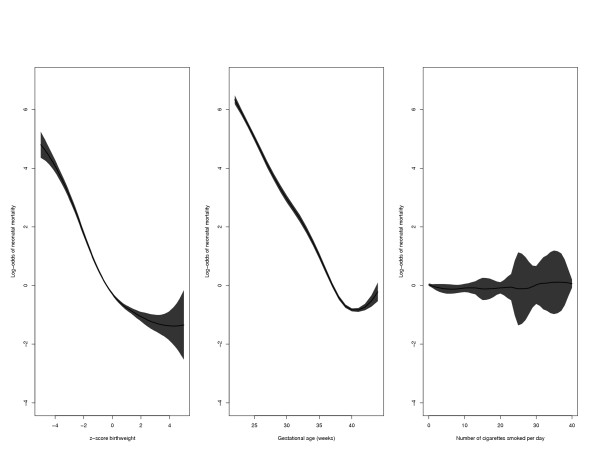
Adjusted log-odds of neonatal mortality (thick curve) with 95 percent point-wise confidence bands (shaded area) for z-score birth weight (left panel), gestational age (middle panel), and number of cigarettes smoked per day (right panel). Each factor was adjusted for the other two factors as well as for maternal age and gravidity.

We first separately examined the effect of each of the 3 covariates standardized birth weight, gestational age, and number of cigarettes smoked per day, on neonatal mortality. This was done by fitting nonparametric logistic regression models (GAM). The univariable GAM strongly suggests that the unadjusted association between standardized z-score birth weight and neonatal mortality is nonlinear (not shown). The association between gestational age and neonatal mortality was also nonlinear, whereas the association between number of cigarettes smoked per day mortality was virtually flat. The adjusted smooth curves for these 3 covariates, along with their corresponding 95% point-wise confidence bands are displayed in Figure 2. These curves were adjusted for the other two factors in addition to maternal age and gravida. It is interesting to note that the relationship between standardized birth weight and neonatal mortality (adjusted for gestational age and smoking and confounders) was virtually flat at increased birth weight z-scores (*i.e*., at z-scores ≥ 4.0).

**Figure 3:**
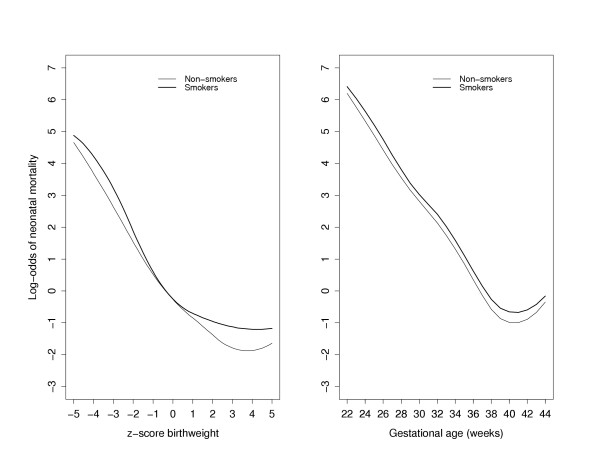
Adjusted log-odds of neonatal mortality based on z-score birth weight (left panel) and gestational age (right panel) among smokers (thick curve) and nonsmokers (thin curve). Each factor was adjusted for the other factor as well as for maternal age and gravidity.

**Figure 4:**
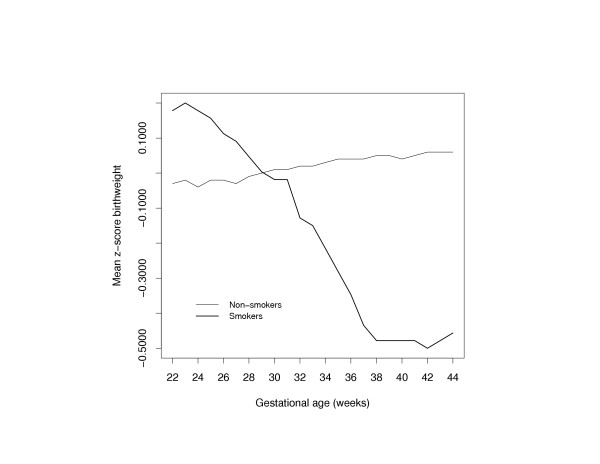
Distribution of gestational age-specific mean z-score birth weight among smokers (thick curve) and nonsmokers (thin curve). The curves were adjusted for maternal age and gravidity.

Since smoking was weakly associated with neonatal mortality, we examined if the effect of smoking on mortality was mediated through either standardized birth weight or gestational age (or both). We therefore modeled neonatal mortality in relation to these 2 covariates (and adjusted for confounders) within broad categories of smokers and nonsmokers (Fig 3). Compared with nonsmokers, neonatal mortality among women that smoked during their pregnancy was higher among infants that were between -5 and -1, and between 1 and 5 standard deviation units of the birth weight distribution among smokers. Infants with birth weight z-scores between -1 and 1 had mortality rates that were similar regardless of maternal smoking status. When neonatal mortality rates were examined by gestational age, the mortality curve was consistently higher at every gestational age among smokers than among nonsmokers (P < 0.001). In order to better understand whether smoking affects fetal growth, we examined the distributions of gestational age-specific standardized birth weight z-scores between the two groups of smokers (Fig 4). The results indicate that the adjusted mean z-score birth weight among nonsmokers is fairly constant across gestational age. However, among women that smoked during pregnancy, the adjusted mean z-score is higher that those of nonsmokers between 22 and 28 weeks, and begins to drop precipitously with increasing gestational age. This pattern indicates that smoking results in more growth restricted infants, and that the effect of smoking on reduced fetal growth appears to get stronger at gestational ages 32 weeks and beyond.

**Figure 5:**
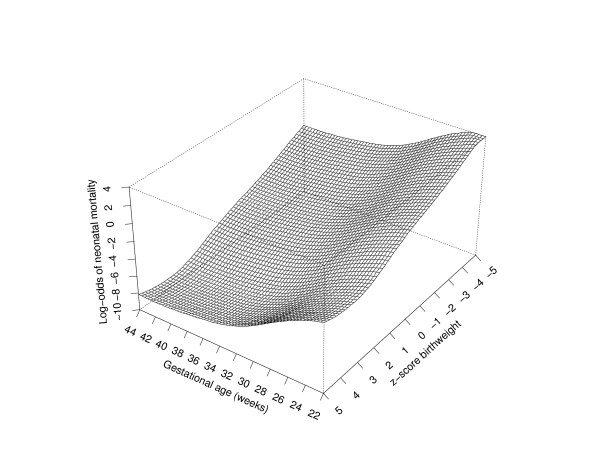
Adjusted smoothed surface of risk of neonatal mortality in relation to z-score birth weight and gestational age. The curve was adjusted for smoking, maternal age, and gravidity.

The logistic regression models discussed thus far are based on the implicit assumption that the combined effects of standardized birth weight and gestational age are multiplicative on a logistic scale. We examined the sensitivity of this assumption by modelling neonatal mortality by allowing an interaction term between these two factors based on nonparametric smooth fit. The joint effect of standardized birth weight and gestational age on neonatal mortality reveals that both reduced fetal growth and early delivery result in increasing mortality risk, with the mortality plane progressively diminishing with increasing standardized birth weight and gestational age (Fig 5).

## Discussion

For decades, several researchers have focused on trying to understand the complex biological relationship among pregnancy duration, infant size, and neonatal mortality. Not only are gestational age and birth weight highly correlated, but both are powerful predictors of neonatal mortality [14-16]. The chief findings from our study include (i) z-score birth weight and preterm delivery (independent of birth weight) exert strong influences on neonatal mortality; (ii) the effect of maternal smoking is mediated largely through reduced fetal growth and, to a smaller extent, through shortened gestation; and (iii) mortality among babies born to smoking mothers is virtually higher at every z-score birth weight (independent of gestational age) than those born to nonsmoking mothers.

The inverted "J"-shaped relationship between birth weight and mortality essentially holds for analyses relating to gestational age and mortality. While birth weight is considered a marker for fetal size, gestational age is thought of as an indicator of fetal maturity. Almost 3 decades ago, Susser and colleagues [15] proposed that gestational age is causally precedent to birth weight (implying that birth weight is in the causal pathway of the gestational age-mortality relationship). Wilcox and Skjaerven [16] examined close to 400,000 singleton births from Norway in an effort to separate the influences of birth weight and gestational age on neonatal mortality. They showed that, comparisons using the "relative birth weight" scale, there were two strong and separable factors related to mortality: gestational age independent of birth weight, and relative birth weight at any given gestational age.

On these similar lines, Herman and Hastie [17] examined neonatal mortality in relation to (absolute) birth weight and gestational age. They initially speculated that among preterm (<37 weeks) babies, maturity would serve as a strong predictor of mortality, while among term babies, the increased mortality was probably due to growth restriction. However, their analysis showed that mortality was associated only with birth weight and not with gestational age. Their approach to analysis may have suffered from collinearity (between birth weight and gestational age), perhaps leading to the attenuated gestational age-mortality relationship [17]. Coory [18] analyzed neonatal mortality in relation to birth weight and gestational age. He concluded that both birth weight and gestational age have independent effects on mortality, and that both are fundamental risk-adjusting variables. However, he was cautious in not interpreting the effects of gestational age, but focused his interpretations almost entirely on birth weight. Our construction of standardized birth weight z-score was developed conditional on gestational age. Thus, this birth weight z-score (independent of gestational age) enabled us to assess the effects of shortened gestation and fetal growth restriction on mortality.

It is widely acknowledged that smoking mothers give birth to infants that are lighter compared with those born to nonsmoking mothers. This reduction in birth weight is thought mainly to result in fetal growth restriction, as well as to shortened gestation [19,20]. Although the precise mechanism by which smoking during pregnancy affects the fetus is unclear, two possible pathways have been proposed. Smoking results in increased capillary fragility and vasoconstriction of arterial walls, leading to reduced blood flow to the uterus and eventually to the placenta [21]. The second is the "fetal hypoxia" hypothesis, whereby smoking leads to a villous shrinkage due to an alteration in the thickness of the villous membrane, thereby reducing oxygen transfer to the fetus [22]. Both mechanisms are likely to increase the risk of uteroplacental bleeding in pregnancy [23], which, in turn, increases the risk of not only neonatal deaths [20,24], but also preterm delivery and growth restriction [23]. Our study provides circumstantial evidence that after the general effects of (shortened) gestational age and (reduced) fetal growth are accounted for, smoking has little direct impact on neonatal mortality.

Our study has some limitations. First, errors in the estimation of gestational age [25,26] are likely to affect our results to some extent. Our study was based on gestational age largely determined from the date of last menstrual period as opposed to one based on early ultrasound. Sonographically estimated gestational age is likely to shift the overall gestational age distribution to lower gestational ages [26] sometimes by as much as a full menstrual cycle [25], possibly due to delayed ovulation or amenorrhea. Second, the impact of congenital malformations and chromosomal abnormalities on the risk of neonatal death could have been partly responsible for the findings noted here. Although data on malformations are contained on the vital statistics files, they are recorded poorly. Third, although we adjusted all the analysis for maternal age and gravidity, the study does not take into account other known or suspected risk factors for neonatal mortality. These risk factors may account for a part of the associations noted here, but is unlikely that these factors could explain the powerful effects of fetal growth restriction and preterm delivery on neonatal mortality. Finally, non-differential misclassification of smoking data on vital records is likely [27] and may have attenuated the smoking-mortality association to some extent.

Application of generalized additive regression models to examine neonatal mortality appears useful towards understanding the complex biological relationship amongst the predictors. However, we make no claim that GAMs serve as adjuncts to other modeling approaches; on the contrary, we believe that GAMs can provide the first step toward modeling complex exposure-disease relationships.

## Conclusions

Our study provides important insights about the combined effects of gestational age, fetal growth, and smoking during pregnancy on neonatal mortality. Both standardized z-score birth weight and preterm delivery are strongly associated with neonatal mortality, and the effect of maternal smoking appears largely mediated largely through reduced fetal growth and, to a smaller extent, through shortened gestation.

## Competing interests

The author(s) declare that there are no competing interests.

## Authors' contributions

CVA and RWP conceived the idea for the study. CVA assembled the data and performed all statistical analyses. CVA drafted the manuscript with essential contributions from RWP. Both authors critically reviewed the manuscript, edited it for content, and revised it to the final form.

Both authors have read and approve the final manuscript.

## Pre-publication history

The pre-publication history for this paper can be accessed here:



## Supplementary Material

Additional File 1 Description of generalized additive models. Click here for file
