# Supplementary material for: Reexamining the effects of gestational age, fetal growth, and maternal smoking on neonatal mortality
Source: BMC Pregnancy Childbirth. 2004 Dec 1;4:22. doi: 10.1186/1471-2393-4-22 (PMC535930; doi:10.1186/1471-2393-4-22)
Supplement: Additional File 1 — Description of generalized additive models. [file 1471-2393-4-22-S1.pdf]

APPENDIX

Generalized additive models

Generalized additive models are a nonparametric analogue of the multivariable logistic regression model. Let Y_i denote a binary response (deaths coded 1, and survivors 0) for the ith subject($1 \leq i \leq n$), and $x_i = (x_{i1}, x_{i2}, \cdots, x_{ip})$ denote a vector of $p-$explanatory variables or predictors of risk. The probability of a positive response, $\mu_{ij} = \Pr(Y_i = 1|x_{ij})$, has a logistic regression structure with the specification

$$\log\left[\frac{\Pr(Y_i = 1|x_{ij})}{1 - \Pr(Y_i = 1|x_{ij})}\right] = \alpha + \sum_{j=1}^{p} \beta_j(x_{ij}) \tag{1}$$

The expression log [.] is the conditional log–odds of the positive response, also known as the "logit" function. The terms β_j is a set of unknown regression coefficients corresponding to the the p-covariates, and α denotes the intercept term. When the right hand side of the regression model is replaced by an additive combination of smooth, but otherwise unspecified functions of the predictors, the model above reduces to

$$\log\left[\frac{\Pr(Y_i = 1|x_{ij})}{1 - \Pr(Y_i = 1|x_{ij})}\right] = \alpha + \sum_{j=1}^{p} s_j(x_{ij}) \tag{2}$$

The terms $s_j(x_{ij})$ (constrained to have intercept 0) are a set of $p-$unspecified nonparametric functions estimated in a flexible manner. Although the model shown above is fully nonparametric, it can accomodate combinations of smooth terms for a subset of the continuous covariates and not for others. For example, model (1) can contain two continuous covariates X_1 and X_2, with X_1 modeled as a smooth function while X_2 modeled as a simple linear term. In the conventional logistic regression model, the terms $\sum s_j(x_{ij})$ in model (1) are replaced by linear terms, $\sum \beta_j x_{ij}$. The GAM can reveal interesting nonlinearities in the effect of x_{ij}, and allows a data–driven specification of the functional form of the exposure–disease relationship.

The smooth functions $s_j(x_{ij})$ in model (1) are estimated using unidimensional smoothers, which are then combined to produce the GAM. Therefore, plotting the smooth function $s_j(x_{ij})$ against x_{ij} can reveal nonlinear relationships. In addition, a $100(1-\alpha)\%$ pointwise confidence bands for $s_j(x_{ij})$ can be constructed using

$$\left\{\hat{s_j}(x_{ij}) - Z_{\alpha/2} \times \hat{\text{se}}[\hat{s_j}(x_{ij})], \quad \hat{s_j}(x_{ij}) + Z_{\alpha/2} \times \hat{\text{se}}[\hat{s_j}(x_{ij})]\right\} \tag{3}$$

where $\text{se}[s_j(x_{ij})]$ refers to the pointwise standard error of the smooth function $s_j(x_{ij})$, and $Z_{\alpha/2}$ is the critical value of the standard normal distribution. The GAM is estimated based on the *Iterative Reweighted Least Squares* procedure.

Iterative Reweighted Least Squares (IRLS) Estimation

We describe the estimation of parameters in the GAM for a single covariate, X, based on extending the principles from a simple logistic regression model. Consider a pair of estimates, $(\hat{\alpha}^0, \hat{\beta}^0)$, with probability $\hat{\mu_i}^0$, derived from the logistic regression model:

$$\log\left[\frac{\hat{\mu_i}^0}{1-\hat{\mu_i}^0}\right] = \hat{\alpha}^0 + \hat{\beta}^0 x_i$$

An adjusted dependent variable, z_i, is constructed as

$$z_i = \hat{\alpha}^0 + \hat{\beta}^0 x_i + \frac{(y_i - \hat{\mu_i}^0)}{\hat{\mu_i^0}(1-\hat{\mu_i^0})}$$

and weights $w_i = \hat{\mu_i}^0(1-\hat{\mu_i}^0)$. The new or updated estimates $(\hat{\alpha}^1, \hat{\beta}^1)$ are obtained by regressing z_i on x_i, with weights w_i. This procedure is iterated until the estimates converge.

In conjunction with the scatterplot smoother (described below) and the IRLS estimation, the GAM is estimated, iteratively, by smoothing z_i

$$z_i = \hat{\alpha}^0 + \hat{s}^0(x_i) + \frac{(y_i - \hat{\mu_i}^0)}{\hat{\mu_i}^0(1-\hat{\mu_i}^0)}$$

on x_i, with weights w_i as defined earlier. Extending this estimation process to multiple covariates can be based on the multidimensional scatterplot smoother.

Scatterplot Smoothers

Estimation in the context of GAM involves scatterplot smoothers, since it is assumed that the dependency of y_i on x_{ij} is a smooth function. An important property of a smoother is its nonparametric nature, in that it does not assume a rigid form for the dependency of y on x_j. Several scatterplot smoothing methods have been developed (see Hastie and Tibshirani's monograph (13) for a review). We briefly describe the locally–weighted regression scatterplot smoother, *loess* (15), that was utilized in this study (although any smoother can be used).

Locally–weighted regression fits a line to a scatterplot based on the dependency of y on x at several points over the range of observed x. For a target point, x_0, *loess* computes a straight line smooth, $s(x_0)$, based on the following four–step algorithm:

1. The k values of x closest to x_0 (called "nearest neighbors") are identified and denoted as $N(x_0)$. The choice of k is controlled through a "span" argument which defines the size of the neighborhood in terms of a fraction of the size of the data.

2. The distance of the furthest near–neighbor from x_0 (*ie.,* distance of the near neighbor most distant from x_0) is obtained as:

$$\Delta(x_0) = \max_{N(x_0)} |x_0 - x_i|$$

3. Weights, w_i, are assigned to each point in $N(x_0)$, based on the tri–cube function

$$W\left(\frac{|x_0 - x_i|}{\Delta(x_0)}\right)$$

where

$$W(u) = \begin{cases} (1-u^3)^3 & \text{if } 0 \le u < 1 \\ 0 & \text{otherwise} \end{cases}$$

4. $s(x_0)$ is the fitted value at x_0, obtained from the least squares fit of y to x, confined to $N(x_0)$ and using weights w_i.

This algorithm in effect fits regression lines to neighborhoods of each point, and estimates the value of the smooth function by the predicted value from the regression line. The

smoothness is determined by the span.

Inference in GAM

Inferences in GAM can be based on the deviance statistic and by examining plots of partial residuals from the fit of GAMs. Hastie and Tibshirani (13) have developed inference tools for the GAMs based on the deviance statistic. The deviance (up to an additive constant) is twice the maximized log likelihood. The model deviance is given as the difference between an intercept–only model with that of a model containing covariates. The deviance therefore can be used to measure the fit of a model relative to other candidate models. Improvement in model fit (or lack thereof) by adding covariates to the model is based on computing effect deviances. The effect deviance is based on computing the difference in deviances between two nested models. Improvement in the fit of a more complex model relative to a simpler model can be based on an approximate χ^2 test, with degrees of freedom (`df`) equal to the difference in the number of covariates contained in the two models being compared. The nonparametric *df* for covariates can take non–integer values, details of which can be found in the monograph by Hastie and Tibshirani (13). Complex models in GAM can therefore be compared with reduced models so long as one model is nested within the other. An analysis of deviance can then be performed to compare nested GAMs.

In the event that models are non–nested, the Akaike Information Criterion (AIC) can be used to compare models. This works as it does in other modeling settings – the deviance is adjusted for the number of parameters in the model.

Once the GAM is fit, residuals, r_{ij}, defined as

$$\hat{r_{ij}} = \frac{(y_i - \hat{\mu_i})}{\hat{\mu_i}(1 - \hat{\mu_i})}$$

can be computed and plotted against each predictor in the GAM. Influence of outlying observations or poorly fitting models can then be assessed from these plots.
